# Identification of a plastid intercistronic expression element (IEE) facilitating the expression of stable translatable monocistronic mRNAs from operons

**DOI:** 10.1111/j.1365-313X.2007.03261.x

**Published:** 2007-12

**Authors:** Fei Zhou, Daniel Karcher, Ralph Bock

**Affiliations:** Max-Planck-Institut für Molekulare Pflanzenphysiologie (MPI-MP) Am Mühlenberg 1, D-14476 Potsdam-Golm, Germany

**Keywords:** chloroplast, RNA processing, intercistronic processing, RNA cutting, polycistronic transcript, plastid transformation

## Abstract

Most plastid genes are part of operons and expressed as polycistronic mRNAs. Many primary polycistronic transcripts undergo post-transcriptional processing in monocistronic or oligocistronic units. At least some polycistronic transcripts are not translatable, and endonucleolytic processing may therefore be a prerequisite for translation to occur. As the requirements for intercistronic mRNA processing into stable monocistronic transcript are not well understood, we have sought to define minimum sequence elements that trigger processing and thus are capable of generating stable translatable monocistronic mRNAs. We describe here the *in vivo* identification of a small intercistronic expression element that mediates intercistronic cleavage into stable monocistronic transcripts. Separation of foreign genes by this element facilitates transgene stacking in operons, and thus will help to expand the range of applications of transplastomic technology.

## Introduction

In plastids (chloroplasts), primary transcripts undergo a complex series of mRNA maturation steps. These include processing of the 5′ and 3′ ends (RNA trimming), intron splicing, RNA editing, and cleavage of polycistronic precursor transcripts into monocistronic or oligocistronic mRNAs (RNA cutting). Most of these RNA processing steps exhibit prokaryotic features that have been retained from the cyanobacterial ancestor of all present-day plastids. 5′ and 3′ end processing in plastids is catalyzed by nucleus-encoded prokaryotic-type ribonucleases. Whereas 5′ end maturation is catalyzed primarily by endoribonucleases, 3′ end formation is mediated by the concerted action of endoribonucleases and 3′→5′ exoribonucleases (Sakamotu *et al.*, 1994; Hayes *et al.*, 1996; reviewed in Monde *et al.*, 2000; Herrin and Nickelsen, 2004). Stem–loop-type RNA secondary structures within the 5′ and 3′ untranslated regions (UTRs) of plastid messenger RNAs provide important recognition elements for RNA processing enzymes, and, in addition, can serve as protective elements preventing rapid RNA degradation (Barkan and Goldschmidt-Clermont, 2000; Mayfield *et al.*, 1995; Monde *et al.*, 2000; Stern and Gruissem, 1987).

As in eubacteria, most genes in plastids are organized in polycistronic transcription units (operons). Transcription of bacterial operons usually gives rise to stable polycistronic mRNAs that are directly translated, although in some cases processing to monocistronic mRNAs is known to occur and is involved in gene regulation (e.g. Carpousis *et al.*, 1989). In plastids, most polycistronic precursor transcripts are post-transcriptionally processed into monocistronic or oligocistronic units, presumably by specific endonucleolytic cleavage (Herrin and Nickelsen, 2004; Sugita and Sugiura, 1996; Westhoff and Herrmann, 1988). One of the exceptions is the *psbE* operon, which comprises four small genes for polypeptides of photosystem II (*psbE*, *psbF*, *psbL* and *psbJ*; Carrillo *et al.*, 1986; Willey and Gray, 1989). The *psbE* operon is transcribed as a single 1.1 kb mRNA species that remains tetracistronic and is not processed further. Other examples of unprocessed polycistronic transcripts include the *psaA/B* transcript (Meng *et al.*, 1988) and *petA*, which represents the last cistron of a large polycistronic transcript and is not cleaved off from the upstream open reading frame *ycf10* (Willey and Gray, 1990). The transcripts from most other plastid operons undergo intercistronic processing (also referred to as RNA cutting; Sugiura, 1992), and, at least in some cases, cutting into monocistronic units is an essential processing step: while some polycistronic precursor transcripts can be translated (Barkan, 1988), others must be processed to become translatable or make translation more efficient. This is supported by the analysis of nuclear mutants defective in distinct intercistronic processing events, as well as by *in vitro* translation studies. For example, the maize *crp1* mutant is defective in intercistronic processing between the *petB* and *petD* cistrons, which results in a concomitant loss of *petD* translation (Barkan *et al.*, 1994; Fisk *et al.*, 1999), suggesting that *petD* needs to be monocistronic to be translated. Similarly, defective processing of *psbH* mRNA from the pentacistronic primary transcript of the *psbB* operon leads to loss of *psbH* translation in the Arabidopsis *hcf107* mutant (Felder *et al.*, 2001). Another Arabidopsis mutant with impaired intercistronic RNA processing is *crr2*, in which endonucleolytic cleavage between the *rps7* and *ndhB* cistrons does not occur (Hashimoto *et al.*, 2003). This results in loss of the NDH complex, most probably because the unprocessed *ndhB* message cannot be translated (Hashimoto *et al.*, 2003). mRNA secondary structure formation has been implicated in impaired translatability of unprocessed polycistronic precursors (Felder *et al.*, 2001; Hirose and Sugiura, 1997). Direct evidence for this has come from *in vitro* translation experiments with *ndhD* transcripts, another plastid mRNA whose translation is dependent on prior intercistronic processing. Translation of the di-cistronic *psaC*–*ndhD* precursor transcript was shown to be impaired by RNA secondary structure formation between a short (8 nt) sequence within the *psaC* coding region and a complementary sequence in the 5′ UTR of the downstream *ndhD* (Hirose and Sugiura, 1997).

As such long-range secondary structural interactions are not easily predictable, it is generally not possible to make educated guesses about the translatability of polycistronic transcripts in plastids. This is highly unfortunate, because simultaneous expression of multiple transgenes from operons is viewed as one of the unique attractions of chloroplast transformation technology (Bogorad, 2000; Daniell and Dhingra, 2002; Heifetz, 2000; Maliga, 2004). Expression of transgenes from polycistronic mRNAs has been successful in some cases (Quesada-Vargas *et al.*, 2005; Staub and Maliga, 1995), but poor translation of polycistronic mRNAs is likely to be responsible for at least some cases where transgene expression was disappointingly low (Nakashita *et al.*, 2001) or unsuccessful altogether (Magee *et al.*, 2004). Clearly, processing of polycistronic transcripts into stable monocistronic mRNAs would greatly reduce the risk of failure of transgene expression from the plastid genome and thus make transplastomic experiments more predictable.

Here, we report a solution for this problem. We have identified a small sequence element, referred to as an intercistronic expression element (IEE), that mediates the efficient intercistronic cleavage of polycistronic mRNAs into stable monocistronic transcripts. We show that, while this element is not required for processing downstream of the first cistron to occur, it is essential to confer mRNA stability and translation of the second cistron. The identified IEE is small enough to serve as a universal tool for stacking of foreign genes in operons, and thus will help to extend the range of applications of transplastomic technology.

## Results

### Mapping of intercistronic mRNA processing sites in the tobacco *psbB* operon transcript

**Figure 1:**
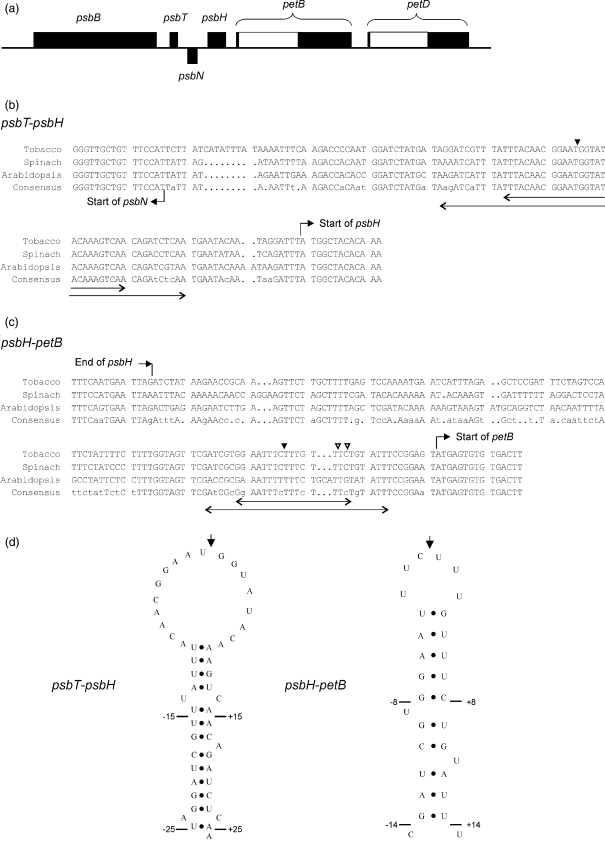
Identification of intercistronic mRNA processing sites in the tobacco *psbB* operon (a) Structure of the *psbB* operon. Genes above the lines are transcribed from left to right; the gene below the line (*psbN*) is transcribed in the opposite direction. The group II introns within the *petB* and *petD* coding regions are shown as open boxes. Transcription from the *psbB* promoter produces a pentacistronic mRNA that undergoes a complex series of processing steps resulting in monocistronic and oligocistronic mRNA species (Westhoff and Herrmann, 1988). (b) Partial sequence alignment of the *psbT*–*psbH* spacer region from tobacco, spinach and Arabidopsis. Shown is the 3′ part of the spacer, between the antisense *psbN* sequence and *psbH*. The intercistronic RNA processing site mapped in tobacco is indicated by a closed triangle. The sequences chosen as putative processing sequences in plastid transformation experiments are indicated by the double-arrowed lines. (c) Alignment of the *psbH*–*petB* spacer regions from tobacco, spinach and Arabidopsis. The major intercistronic RNA processing site mapped in tobacco is marked by a closed triangle; additionally identified minor processing sites are indicated by open triangles. (d) Location of intercistronic processing sites within putative RNA stem–loop structures. The major endonucleolytic cleavage sites are indicated by arrowheads.

To identify sequence elements suitable for triggering processing of polycistronic transcripts into stable and translatable monocistronic mRNAs, we analyzed processing in the tobacco *psbB* operon (Figure 1a), which is one of the best characterized multi-gene operons in plastids (Felder *et al.*, 2001; Meierhoff *et al.*, 2003; Westhoff and Herrmann, 1988). The *psbB* operon consists of five genes, three of which encode photosystem II components (*psbB*, *psbT* and *psbH*), with the remaining two encoding subunits of the cytochrome b_6_f complex (*petB* and *petD*) (Figure 1a). The five genes are co-transcribed, giving rise to a long pentacistronic precursor RNA, which is then cleaved into smaller units by a complex series of processing events (Westhoff and Herrmann, 1988). Not all final processing products are monocistronic: the small *psbT* cistron remains associated with the upstream *psbB*, forming a di-cistronic mature mRNA, and the two cytochrome b_6_f components, *petB* and *petD*, are only inefficiently processed into monocistronic mRNAs, leaving a large proportion of the transcripts di-cistronic (Felder *et al.*, 2001; Westhoff and Herrmann, 1988). Stem–loop-type secondary structures are found upstream of most cleavage sites, suggesting that they stabilize the 3′ ends of the mature transcripts (Stern and Gruissem, 1987; Westhoff and Herrmann, 1988).

We decided to map the intercistronic cleavage sites upstream and downstream of the *psbH* cistron in tobacco, because *psbH* is efficiently cleaved into a monocistronic mRNA by two endonucleolytic cleavage events upstream and downstream (Felder *et al.*, 2001; Westhoff and Herrmann, 1988). To precisely identify the cleavage sites, we employed an RNA circularization-based method by which the head-to-tail ligated 5′ and 3′ UTRs of the mRNA can be simultaneously analyzed (Zandueta-Criado and Bock, 2004). This analysis revealed a major cleavage site upstream of *psbH* (Figure 1b), two nucleotides away from the suggested processing site in Arabidopsis that was determined by S1 nuclease mapping (Felder *et al.*, 2001). In the *psbH*–*petB* intergenic spacer, we identified one major and two minor cleavage sites (Figure 1c). The major site was found in four of the six clones sequenced, the minor sites in one clone each.

We next wished to determine whether RNA secondary structures are potentially involved in cleavage, for example whether they could mediate cleavage site recognition by a specific endoribonuclease. We therefore analyzed the nucleotide sequences surrounding the identified cleavage sites for their potential to fold into stable secondary structures. This was the case for both the processing site upstream and the site downstream of *psbH* (Figure 1d). Interestingly, in both structures, the cleavage site is localized approximately in the middle of the central loop of a stem–loop structure, possibly suggesting that cleavage site selection is aided by the formation of RNA secondary structures.

### Integration of transgene operons with putative processing elements into the tobacco plastid genome

**Figure 2:**
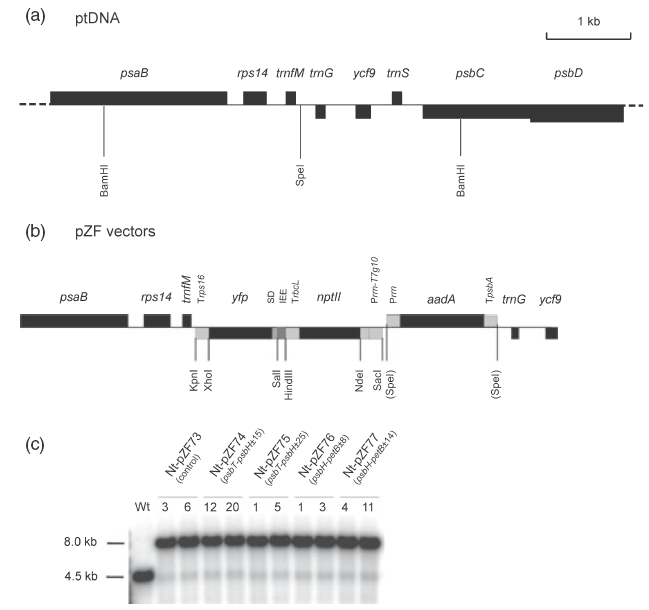
Generation of plastid-transformed plants to test putative intercistronic processing elements *in vivo* (a) Map of the targeting region in the tobacco plastid genome (ptDNA). Genes above the lines are transcribed from left to right; genes below the line are transcribed in the opposite direction. The transgenes are targeted to the intergenic spacer between the *trnfM* and *trnG* genes. (b) Construction of plastid transformation vectors (pZF series) integrating an operon of two transgenes (*nptII* and *yfp*) into the tobacco plastid genome, along with the selectable marker gene for chloroplast transformation, *aadA* (Svab and Maliga, 1993). Relevant restriction sites used for cloning and RFLP analysis are indicated, sites lost by ligation of heterologous ends are shown in parentheses. Prrn, rRNA operon promoter; T*psbA*, 3′ UTR of the *psbA* gene; P*rrn-T7g10*, rRNA operon promoter fused to the leader sequence of bacteriophage T7 gene 10; T*rbcL*, 3′ UTR of the *rbcL* gene; IEE (putative processing element); SD, Shine–Dalgarno sequence; Trps16, 3′ UTR of the *rps16* gene. (c) Southern blot confirming plastid transformation and assessing homoplasmy of transplastomic lines. Digestion with *Bam*HI produces fragments of approximately 4.5 kb in the wild-type and approximately 8 kb in all transplastomic lines. This size difference corresponds exactly to the combined size of the three integrated transgenes. Note that the probe (a radiolabeled *ycf9* fragment) detects a faint wild-type-like band in all transplastomic lines that has been shown previously to come from promiscuous chloroplast DNA in the tobacco nuclear (or mitochondrial) genome (Ruf *et al.*, 2000).

To identify a minimum sequence element sufficient for triggering processing of polycistronic transcripts into stable and translatable monocistronic mRNAs, we decided to test sequences derived from the two major processing sites mapped upstream and downstream of *psbH in vivo* by chloroplast transformation. To this end, we constructed a plastid transformation vector with two transgenes linked together in an operon: the kanamycin resistance gene *nptII* and the gene for the yellow fluorescent protein, *yfp* (Figure 2a,b). The two coding regions are separated by a sequence encoding a stem–loop structure (*TrbcL*) (Figure 2b) to ensure transcript stability of the mRNA from the first cistron after processing, two restriction sites suitable for integrating potential intercistronic processing elements, and a Shine–Dalgarno sequence to mediate translation initiation at the second cistron. For both major processing sites (Figure 1b–d), we constructed two chloroplast transformation vectors (Table 1). Vectors pZF75 and pZF77 contain the complete secondary structures in which the cleavage sites are embedded. This corresponds to sequence elements from −25 to +25 with respect to the *psbT*–*psbH* processing site (Figure 1b,d; vector pZF75) and −14 to +14 with respect to the *psbH*–*petB* processing site (Figure 1c,d; vector pZF77). In addition, we used two shorter sequences that included only the stem–loop up to the first bulge in the stem structure (Figure 1d). These sequence elements correspond to nucleotide positions −15 to +15 with respect to the *psbT*–*psbH* processing site (Figure 1b,d; vector pZF74) and −8 to +8 with respect to the *psbH*–*petB* processing site (Figure 1c,d; vector pZF76). Finally, a fifth construct containing no putative processing element between the *nptII* and *yfp* cassettes was transformed as a control (pZF73; Table 1).

**Table 1:** Features of chloroplast transformation vectors and transplastomic lines generated in this study

Vector	Transplastomic lines	Presence of a potential intercistronic expression element (IEE)	Origin of the potential IEE (intergenic spacer)	Size of the potential IEE
pZF73	Nt-pZF73	−	−	0
pZF74	Nt-pZF74	+	*psbT*–*psbH*	±15
pZF75	Nt-pZF75	+	*psbT*–*psbH*	±25
pZF76	Nt-pZF76	+	*psbH*–*petB*	±8
pZF77	Nt-pZF77	+	*psbH*–*petB*	±14

The constructs were introduced into the tobacco plastid genome by biolistic chloroplast transformation (Svab and Maliga, 1993). Two homologous recombination events in the regions flanking the three transgenes (the *nptII*–*yfp* operon and the selectable spectinomycin resistance gene *aadA*) (Figure 2a,b) incorporate the foreign genes into the plastid genome. Selection of bombarded leaf samples for resistance to spectinomycin conferred by the *aadA* marker gene yielded several transplastomic lines that were subjected to additional rounds of regeneration and selection to obtain homoplasmic tissue (Bock, 2001; Svab and Maliga, 1993). After three such rounds, plants were regenerated, rooted in sterile culture, transferred to soil and grown to maturity in the glasshouse. Plants from all transplastomic lines were phenotypically identical in that they were indistinguishable from wild-type plants (not shown). For each construct, two independently generated lines were selected for further analysis. The transplastomic lines will be subsequently referred to as Nt-pZF followed by the number of the construct and the number of the individual transplastomic line (e.g. Nt-pZF73-3, indicating transplastomic tobacco line number 3 generated using construct pZF73).

To confirm correct integration of the transgenes into the plastid genome and to test for homoplasmy of the transplastomic lines, RFLP analyses were performed (Figure 2c, and data not shown). These assays revealed, in addition to a strong band of the expected size for the transplastomic fragment, a faint hybridization signal that corresponded in size to the restriction fragment from the wild-type genome (Figure 2c). Persistence of a wild-type-like hybridization signal even after multiple rounds of selection and regeneration is often seen in transplastomic lines and usually is not caused by true heteroplasmy of the plastid transformants, but rather by the presence of promiscuous plastid DNA in one of the other two genomes of the plant cell. This is because, during evolution, large fragments of plastid DNA have integrated into the nuclear and mitochondrial genomes (for review, see Bock, 2006; Timmis *et al.*, 2004) as non-functional, so-called ‘promiscuous DNA’. Our previous work has established that the wild-type-like bands in DNA gel-blot analyses of otherwise homoplasmic transplastomic lines originate from such promiscuous DNA (Figure 2c) (Hager *et al.*, 1999; Ruf *et al.*, 2000).

**Figure 3:**
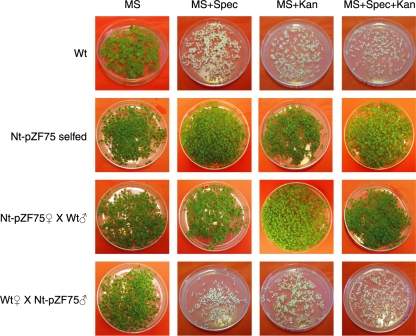
Seed assays to confirm homoplasmy of Nt-pZF transplastomic plants and test for antibiotic resistance Seeds from the wild-type, a selfed transplastomic line and reciprocal crosses between the transplastomic line and the wild-type were germinated on antibiotic-free medium, medium with spectinomycin (Spec; 500 mg l^–1^), medium with kanamycin (Kan; 400 mg l^–1^) and medium with both drugs (Spec + Kan). Maternal inheritance of both antibiotic resistances confirms transgene localization in the plastid genome; lack of segregation in the T_1_ generation confirms homoplasmy of the transplastomic lines.

To ultimately confirm homoplasmy of the transplastomic lines, seeds were obtained from transplastomic plants that had either been selfed or reciprocally crossed to the wild-type. With these seeds, inheritance assays were performed, which represent the most sensitive available test to assess homoplasmy (Bock, 2001; Maliga, 2004). As expected, lack of segregation of spectinomycin resistance in the T_1_ generation demonstrated homoplasmy (Figure 3, and data not shown) and confirmed uniparentally maternal transgene inheritance, as is typical of a plastid-encoded trait.

### Analysis of transcript accumulation and RNA processing in transplastomic lines

**Figure 4:**
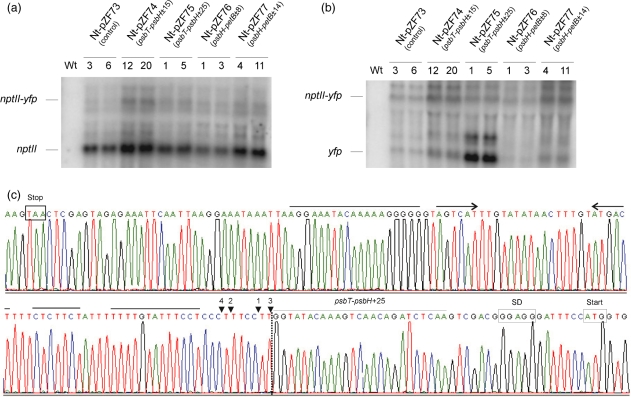
RNA accumulation in transplastomic lines harboring various candidate processing elements between the *nptII* and *yfp* cistrons (a) Accumulation of *nptII* mRNA. All transplastomic lines accumulate predominantly monocistronic *nptII* mRNA, in addition to small amounts of di-cistronic *nptII*–*yfp* transcripts and other minor RNA species that were not further characterized. Accumulation of monocistronic *nptII* message in the Nt-pZF73 control lines demonstrates that 3′ processing of *nptII* mRNA is independent of the presence of the putative processing sequences. (b) Accumulation of *yfp* mRNA. Significant amounts of (monocistronic) *yfp* mRNA accumulated only in the Nt-pZF75 lines that harbor the ±25 IEE from the *psbT*–*psbH* intergenic spacer. Note that, in addition to the monocistronic *yfp* and di-cistronic *nptII*–*yfp* transcripts, two minor RNA species also accumulate. One of them is approximately 200 bp larger than the monocistronic *yfp* message, and the other is approximately 200 bp larger than the di-cistronic *nptII*–*yfp* transcript. These minor RNA species were not further characterized, but the most probable explanation is that they originate from read-through transcription through *trnfM* (see Figure 1b), whose antisense transcript can also fold into a stable cloverleaf-like secondary structure and thus act as an RNA processing signal. (c) Mapping of the 5′ and 3′ ends of the monocistronic *yfp* mRNA in Nt-pZF75 plants. The sequence of a cDNA clone derived from head-to-tail ligated *yfp* mRNA is shown. The ligation site (i.e. the border between the 3′ end and the 5′ end of the circularized mRNA) is indicated by the dotted vertical line. Note that the 5′ end generated by processing within the *psbT*–*psbH* spacer element is identical in all ten cDNA clones and corresponds precisely to the 5′ end of the *psbH* mRNA (Figure 1b,d). Alternative 3′ ends are indicated by arrowheads, and the number of clones in which the respective termini were found is indicated. A putative transcript-stabilizing stem–loop-type RNA secondary structure within the *rps16* 3′ UTR is marked by horizontal arrows (interruptions indicate unpaired nucleotides). The stop codon, Shine–Dalgarno sequence and start codon are boxed.

Having successfully generated homoplasmic transplastomic plants with all vectors, we next wished to compare the five constructs with respect to RNA processing and transcript accumulation for the two genes of the operon. We first analyzed transcript pattern and RNA accumulation for *nptII*, the first cistron of the operon. Surprisingly, when RNA gel blots were hybridized to an *nptII*-specific probe, identical transcript patterns were detected in all transplastomic lines (Figure 4a): a strongly hybridizing band corresponding in size to the monocistronic *nptII* message was seen in all lines. In addition, weakly hybridizing larger RNA species were detected, including a transcript of the size of the di-cistronic *nptII*–*yfp* RNA (Figure 4a). The same transcript pattern was also present in the Nt-pZF73 control lines that do not harbor a putative processing element, indicating that processing downstream of *nptII* does not require a specific sequence element. This may suggest that the transcript-stabilizing stem–loop structure downstream of the *nptII* coding region (taken from the *rbcL* 3′ UTR) (Figure 2b) is sufficient to mediate faithful 3′ end formation of the *nptII* mRNA.

Next, we investigated transcript pattern and mRNA accumulation of *yfp*, the second cistron of the operon. Interestingly, high amounts of monocistronic *yfp* mRNA accumulated only in the Nt-pZF75 lines harboring the complete stem–loop structure surrounding the *psbT*–*psbH* intercistronic processing site. All other lines had at best small amounts of *yfp* mRNA that corresponded in size to monocistronic message (Figure 4b). Remarkably, lack of accumulation of monocistronic *yfp* mRNA in these lines was not accompanied by increased accumulation of the di-cistronic precursor RNA. This, together with the presence of comparable amounts of monocistronic *nptII* mRNA in all lines, suggests that the presence of a putative processing element does not influence formation of the *nptII* 3′ end. Instead, it appears to serve as a critical mRNA stability determinant for the second cistron, *yfp* (Figure 4a,b).

In order to exclude the possibility that intercistronic processing in the Nt-pZF75 lines did not occur within the *psbT*–*psbH* intercistronic processing site, but instead was caused by sequence context-dependent processing at the upstream T*rbcL* sequence (Figure 2b) (Chakrabarti *et al.*, 2006; Staub and Maliga, 1995), we mapped the mRNA 5′ end of the monocistronic *yfp* mRNA. RNA circularization and analysis of the head-to-tail ligated 5′ and 3′ UTRs allowed us to determine the termini of both mRNA ends. Sequencing of ten individual clones revealed that, in all cases, 5′ processing occurred faithfully within the *psbT*–*psbH* intercistronic processing element, confirming that this sequence element indeed serves as an intercistronic processing signal triggering the cleavage of polycistronic into monocistronic mRNAs. All ten clones had an identical 5′ end to the *psbH* mRNA (Figures 1b and 4c). In contrast, the 3′ end was slightly variable, as has been observed previously for many other plastid mRNAs. All 3′ ends mapped 3–10 nucleotides downstream of a putative transcript-stabilizing stem–loop structure within the *rps16* 3′ UTR of the chimeric *yfp* (Figure 4c).

### Accumulation of NptII and YFP proteins expressed from operon constructs

**Figure 5:**
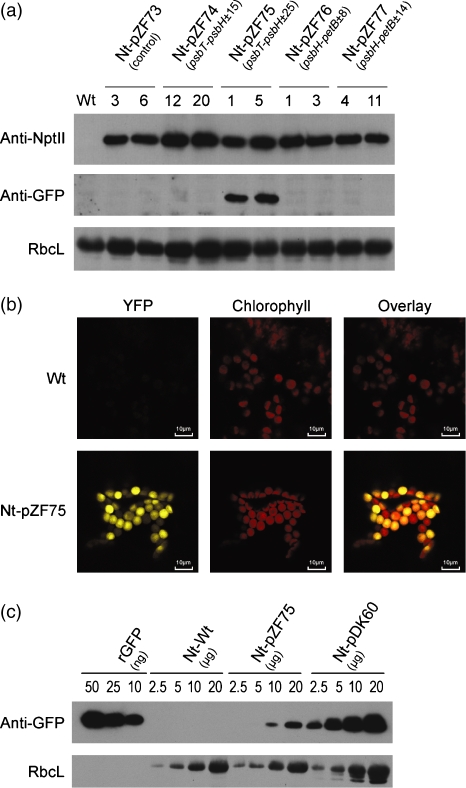
Foreign protein accumulation in transplastomic lines harboring various candidate processing elements between the *nptII* and *yfp* cistrons (a) Western blot detection of NptII and YFP protein accumulation in transplastomic lines. Total soluble proteins were separated by denaturing gel electrophoresis, blotted and probed with anti-NptII or anti-GFP antibodies. As a loading control, accumulation of the large subunit of Rubisco (RbcL) is shown. Whereas the NptII protein accumulates to comparable levels in all transplastomic lines, YFP accumulation is restricted to Nt-pZF75 lines (containing the ±25 IEE from the *psbT*–*psbH* intergenic spacer), correlating with accumulation of stable monocistronic *yfp* message only in these lines. (b) Detection of YFP fluorescence in Nt-pZF75 lines by confocal laser-scanning microscopy. Yellow YFP fluorescence, red fluorescence of the chlorophyll, and the overlay of the two fluorescences are shown for the wild-type and an Nt-pZF75 transplastomic line. (c) Comparison of YFP accumulation in Nt-pZF75 transplastomic lines with GFP accumulation in control transplastomic plants expressing GFP from the P*rrn*–T*rps16* expression cassette (Nt-pDK60). The GFP signal in Nt-pDK60 plants is stronger than the YFP signal in Nt-pZF75 lines, which may be due to weaker recognition of YFP by the anti-GFP antibody and/or lower stability of YFP in plastids. A dilution series with purified GFP protein (rGFP) is also shown. Immunological detection of the Rubisco large subunit (RbcL) was performed as a loading control.

Having established that monocistronic *nptII* transcripts accumulate in all transplastomic lines, whereas stable monocistronic *yfp* mRNA accumulates only in the Nt-pZF75 lines, we next wished to investigate the correlation between RNA abundance and protein accumulation. The high-level kanamycin resistance of all transplastomic lines tentatively indicated that NptII protein accumulates to reasonably high levels (Figure 3, and data not shown). This was confirmed by Western blot analysis with a specific anti-NptII antibody: all lines accumulated similarly high levels of NptII protein (Figure 5a), as expected from the accumulation of similar amounts of monocistronic *nptII* message in all transplastomic lines (Figure 4a). In contrast, when the blots were probed with an anti-GFP antibody (which also recognizes YFP, because YFP is a mutant GFP variant), protein accumulation was only detected in the Nt-pZF75 lines, correlating with the accumulation of stable monocistronic mRNA only in these lines. We therefore conclude that the complete stem–loop structure (±25) surrounding the *psbT*–*psbH* intercistronic processing site (as present in the Nt-pZF75 lines) represents a suitable sequence element to confer stable expression of downstream cistrons in multi-gene operons, and thus can serve as a genuine IEE.

Expression of *yfp* in the Nt-pZF75 lines, but not in the other transplastomic lines, was finally confirmed by confocal laser-scanning microscopy (Figure 5b, and data not shown). When YFP accumulation was compared with GFP accumulation in transplastomic control plants harboring a *gfp* gene under the control of the ribosomal RNA operon promoter, the GFP signal in the control plants was significantly stronger than the YFP signal in the Nt-pZF75 lines (Figure 5c). This may be due to weaker recognition of YFP by the anti-GFP antibody and/or lower stability of YFP in plastids.

## Discussion

Stacking of multiple transgenes is often desirable in both basic research and plant biotechnology. As most plastid genes are arranged in operons and expressed as polycistronic transcripts, easy transgene pyramiding by co-expression of genes from operons is considered to be an attractive advantage of chloroplast transformation over conventional nuclear transformation. Therefore, understanding the rules that govern the efficient expression of multiple linked (trans)genes is a prerequisite for the successful engineering and rational design of complex operons. Earlier work had established that, unlike in eubacteria, post-transcriptional processing of polycistronic mRNAs into monocistronic units is often required for gene expression in plastids (Barkan *et al.*, 1994; Fisk *et al.*, 1999; Hashimoto *et al.*, 2003; Hirose and Sugiura, 1997). However, very little is known about the signals at the RNA level that trigger intercistronic processing into stable and translatable monocistronic mRNAs.

In this work, we have attempted to identify a minimum sequence element, referred to as an IEE, that is suitable to direct cleavage of a polycistronic precursor transcript into stable monocistronic units. We have mapped two intercistronic cleavage sites in the tobacco *psbB* operon and found that they are located in the central position of the loop domain of a putative stem–loop-type RNA secondary structure. *In vivo* testing of these elements in a synthetic operon of two transgenes revealed that only the complete stem–loop structure surrounding the *psbT*–*psbH* processing site conferred expression of the downstream cistron and thus provides a functional IEE. Surprisingly, inclusion of an IEE between the two cistrons of the operon was not required to trigger generation of monocistronic mRNA for the 5′ cistron (*nptII*) (Figure 4), indicating that the stem–loop structure present at the 3′ end of essentially all mature mRNAs in plastids is sufficient to trigger faithful mRNA 3′ end formation of the first cistron of the operon. However, RNA accumulation for the second cistron of the operon turned out to be critically dependent on the presence of a functional IEE. The most probable explanation for this is that, in the absence of a functional IEE, RNA processing does not result in the generation of a stable 5′ UTR of the downstream cistron. It is well established that the 5′ UTR and faithful mRNA 5′ end maturation are critical determinants of mRNA stability in plastids (Nickelsen, 1999; [Bibr b35][Bibr b36]). Therefore, it seems possible that, in the Nt-pZF74, Nt-pZF76 and Nt-pZF77 plants, the intercistronic processing site was not properly recognized, most likely because the *cis*-acting sequence elements required for processing site selection were not completely present. This in turn may have resulted in the usage of aberrant cleavage sites, giving rise to aberrant mRNA species and/or mRNAs with unstable 5′ UTRs that are condemned to rapid degradation (Herrin and Nickelsen, 2004; Monde *et al.*, 2000). This would have no effect on RNA accumulation of the first cistron, *nptII*, because the stable stem–loop structure in its 3′ UTR protects the mRNA from exoribonucleolytic degradation (Hayes *et al.*, 1996, 1999).

The IEE identified in the course of this work provides a novel tool for transgene expression from the chloroplast genome. It simplifies the expression of multiple transgenes by allowing them to be linked in operons, thus eliminating the need to drive each transgene by its own promoter. Promoters for plastid transgenes are usually 100–200 bp fragments taken from endogenous chloroplast genes. Their usage for transgenes duplicates these sequences in the chloroplast genome, and is associated with an increased risk of unwanted homologous recombination resulting in partial genome deletions (Iamtham and Day, 2000; Rogalski *et al.*, 2006). Thus, keeping the number of promoters to a minimum is highly desirable, and it also greatly simplifies vector construction.

The identified IEE is small enough (50 bp) to be integrated in an operon even in multiple copies (if more than two transgenes are to be co-expressed), because below 100 bp homologous recombination in chloroplasts becomes extremely infrequent (Dauvillee *et al.*, 2004; Iamtham and Day, 2000). Separation of the cistrons of an operon by the IEE eliminates the risk of polycistronic mRNAs not being translatable, and therefore will contribute to increasing the success rate of transgene expression from the plastid genome. Potential applications of the IEE include the co-expression of selectable marker and reporter genes, as well as the engineering of complex biochemical pathways (Ye *et al.*, 2000), the introduction of multiple disease resistances, and the production of biopharmaceuticals in plastids (Bock, 2007).

## Experimental procedures

### Plant material and growth conditions

Tobacco plants (*Nicotiana tabacum* cv. Petit Havana) for chloroplast isolation were grown under standard greenhouse conditions. Sterile tobacco plants were grown on agar-solidified MS medium containing 30 g l^–1^ sucrose (Murashige and Skoog, 1962). Regenerated shoots from transplastomic lines were rooted and propagated on the same medium. Rooted homoplasmic plants were transferred to soil and grown to maturity in the glasshouse under standard conditions.

### RNA extraction and circularization, cDNA synthesis and PCR

Chloroplast isolation and RNA extraction from purified chloroplasts were carried out as described previously (Bock, 1998). Total chloroplast RNA (10 μg) was self-ligated at 37°C for 1 h with 20 units of T4 RNA ligase (New England Biolabs; http://www.neb.com) in a final reaction volume of 100 μl. A 1 μg aliquot of the circularized RNA and 38 ng of oligonucleotide PpsbH-RT (5′-TTCCCCACCCAGGAGCTAC-3′) were denatured at 70°C for 5 min and used for cDNA synthesis, which was performed as described previously (Zandueta-Criado and Bock, 2004). The resulting cDNA was used directly as a template for PCR reactions, and amplified according to standard protocols (30 sec at 93°C, 90 sec at 52°C, 90 sec at 72°C; 30 cycles). Primers PpsbH-p1 (5′-AAATCTCCTACCGCAGTTC-3′) and PpsbH-p2 (5′-TGGAGATTTATAATTCTTC-3′) were used for specific amplification of the head-to-tail ligated 5′ and 3′ UTRs. The mRNA 5′ and 3′ ends of the monocistronic *yfp* mRNA were mapped by the same method using 10 μg total RNA from an Nt-pZF75 plant and 38 ng of oligonucleotide Pyfp-RT (5′-CGGTGGTGCAGATGAACTTC-3′) for priming of the cDNA synthesis. Primers Pyfp-p1 (5′-TTGTGGCCGTTTACGTCGCC-3′) and Pyfp-p2 (5′-ACATGGTCCTGCTGGAGTTC-3′) were used for the specific amplification of head-to-tail ligated *yfp* transcripts.

### Cloning and DNA sequencing

Amplified PCR products were cloned into the pCR2.1-TOPO vector using the TOPO TA cloning kit (Invitrogen, http://www.invitrogen.com/). Individual clones were sequenced using M13 reverse primer (MWG-BIOTECH AG; http://www.mwg-biotech.com).

### Construction of plastid transformation vectors

The plastid transformation vectors constructed in this study are based on the previously described vector pRB95 (Ruf *et al.*, 2001). To facilitate analysis of intercistronic processing, two reporter genes were introduced into the polylinker of pRB95: *nptII* and *yfp*. The *nptII* gene is driven by the tobacco plastid rRNA operon promoter (P*rrn*) fused with the 5′ UTR of gene 10 from *Escherichia coli* phage T7 and the downstream box (Kuroda and Maliga, 2001). The terminator is derived from *rbcL* (Kuroda and Maliga, 2001). The *yfp* gene was fused with the Shine–Dalgarno sequence from the *rbcL* gene, and is terminated by the *rps16* terminator (Staub and Maliga, 1994; Wurbs *et al.*, 2007). Between these two genes, various putative processing sequences were integrated (Figures 1 and 2). The coding region of *yfp* was PCR-amplified from plasmid pEYFP-C1 (Clontech, http://www.clontech.com/) using primers Pyfp5′ (5′-TTTTGTCGACG*GGAGG*GATTTCCATGGAGCAAGGGCGAGGAGC-3′) and Pyfp3′ (5′-TTTTCTCGAGTTACTTGTACAGCTCGTCCATG-3′). With these primer sequences, the *rbcL* leader sequence containing the Shine–Dalgarno sequence (in italics in the Pyfp5′ sequence) and the restriction sites *Sal*I and *Xho*I (underlined sequences) were introduced into the PCR product. The resulting amplification product was digested with *Sal*I and *Xho*I, and ligated into the corresponding sites in the polylinker of a pBluescript vector, generating plasmid pBSyfp. The *rps16* terminator was cloned by PCR amplification from tobacco DNA using the primer pair Prps16-5′ (5′-TTTTCTCGAGTAGAGAAATTCAATTAAGG-3′) and Prps16-3′ (5′-TTTTGGTACCCAATTCAATGGAAGCAATG-3′) and introducing*Xho*I and *Kpn*I restriction site (underlined) into the PCR product. The PCR product was digested with the enzymes *Xho*I and *Kpn*I, and cloned downstream of the *yfp* coding region into vector pBSyfp, generating plasmid pBSyfpT. Subsequently, the *nptII* expression cassette from pHK20 (Kuroda and Maliga, 2001) was cloned into the polylinker of pBSyfpT as a *Sac*I/*Hin*dIII fragment. Finally, the two expression cassettes were excised from the pBluescript vector as a 2.2 kb *Sac*I/*Kpn*I fragment, and ligated into the similarly digested plastid transformation vector pRB95, producing vector pZF73. The four different putative processing sequences were inserted into *Hin*dIII/*Sal*I-digested pZF73 as annealed complementary synthetic oligonucleotides that contained compatible single-stranded overhangs for ligation into *Hin*dIII and *Sal*I sites. The following oligonucleotides were used (compatible overhangs are underlined): P5′psbH±15-5′, 5′-AGCTTATTTACAACGGAATGGTATACAAAGTCAAG-3′; P5′psbH±15-3′, 5′-TCGACTTGACTTTGTATACCATTCCGTTGTAAATA-3′; P5′psbH±25-5′, 5′-AGCTTAGGATCGTTTATTTACAACGGAATGGTATACAAAGTCAACAGATCTCAAG-3′; P5′psbH±25-3′, 5′-TCGACTTGAGATCTGTTGACTTTGTATACCATTCCGTTGTAAATAAACGATCCTA-3′; P3′psbH±8-5′, 5′-AGCTTGGAATTTCTTTGTTTCG-3′; P3′psbH±8-3′, 5′-TCGACGAAACAAAGAAATTCCA-3′; P3′psbH±14-5′, 5′-AGCTTGATCGTGGAATTTCTTTGTTTCTGTATTG-3′; P3′psbH±14-3′, 5′-TCGACAATACAGAAACAAAGAAATTCCACGATCA-3′.

### Plastid transformation and selection of homoplasmic transformed tobacco lines

Young leaves from sterile tobacco plants were bombarded with plasmid-coated 0.6 μm gold particles using a PDS1000He biolistic gun (Bio-Rad, http://www.bio-rad.com/). Primary spectinomycin-resistant lines were selected on regeneration medium containing 500 mg l^–1^ spectinomycin (Svab and Maliga, 1993). Spontaneous spectinomycin-resistant plants were eliminated by double selection on medium containing spectinomycin and streptomycin (500 mg l^–1^ each) (Bock, 2001; Svab and Maliga, 1993). For each transformation construct, several independent transplastomic lines were subjected to three to four additional rounds of regeneration on spectinomycin-containing medium to enrich the transplastome and select for homoplasmic tissue.

### Isolation of nucleic acids and hybridization procedures

Total plant DNA was isolated from fresh leaf tissue by a rapid cetyltrimethylammoniumbromide-based mini-prep procedure (Doyle and Doyle, 1990). RNA was extracted using peqGOLD TriFast™ reagent (Peqlab; http://www.peqlab.com) according to the manufacturer’s protocol. For Southern blot analysis, DNA samples (5 μg total DNA) were digested with the restriction enzyme *Bam*HI, separated by gel electrophoresis on 0.8% agarose gels, and transferred onto Hybond XL membranes (Amersham, http://www5.amershambiosciences.com/) by capillary blotting using standard protocols. A 283 bp PCR product generated by amplification of the *ycf9* coding region using primers Pycf9a (5′-GCTGATAGAGGGATCAAAT-3′) and Pycf9b (5′-GGGTCATTTTGGTTTTGGG-3′) was used as an RFLP probe to verify chloroplast transformation. Total cellular RNA samples (10 μg total RNA) were electrophoresed in formaldehyde-containing 1% agarose gels and blotted onto Hybond XL membranes. For detection of *nptII* and *yfp* transcripts, the complete coding regions of the respective genes were excised from plasmid clones. All hybridization probes were purified by agarose gel electrophoresis following extraction of the DNA fragments of interest from excised gel slices using the Nucleospin Extract II kit (Macherey-Nagel; http://www.macherey-nagel.com). Probes were radiolabeled with ^32^P-dCTP using the MegaPrime kit (Amersham). Hybridizations were performed at 65°C in Rapid-Hyb buffer (Amersham) according to the manufacturer’s protocol.

### Protein extraction and immunoblot analyses

Total soluble protein was extracted from leaf samples homogenized in a buffer containing 50 mm HEPES-KOH (pH 7.5), 10 mm KAc, 5 mm MgAc, 1 mm EDTA, 1 mm DTT, 2 mm PMSF and 1%β-mercaptoethanol. Samples representing 10 μg of extracted proteins were separated by electrophoresis in 15% SDS–polyacrylamide gels, and subsequently transferred to polyvinylidene fluoride membranes (Amersham). NptII protein was detected with a specific anti-NptII antibody generated in rabbits (Linaris GmbH; http://www.linaris.de), and the GFP protein was detected with a monoclonal mouse anti-GFP antibody (JL-8, Clontech). Immunobiochemical detection was performed using the ECL Plus detection system (Amersham) according to the manufacturer’s instructions. Purified recombinant GFP protein (rGFP, BD Biosciences; http://www.bdbiosciences.com) served as a standard.

### Microscopy

Subcelluar localization of YFP fluorescence was determined by confocal laser scanning microscopy (TCS SP2; Leica; http://www.leica.com) with an argon laser (488 nm). YFP fluorescence was visualized using a 514 nm excitation and a 527 nm emission filter. Chlorophyll fluorescence was detected using a 670–750 nm filter. All images were acquired using a 63 x objective lens.
